# Trends and Comparisons of Utilization of Emergency Departments Due to Traumatic or Non-Traumatic Causes among the HIV-Positive Population in Taiwan, 2006–2011

**DOI:** 10.3390/ijerph14101214

**Published:** 2017-10-11

**Authors:** Ching-Heng Lin, Ting Lin, Pesus Chou, Nan-Ping Yang

**Affiliations:** 1 Community Medicine Research Center, School of Medicine, National Yang-Ming University, Taipei 11221, Taiwan; epid@ms39.hinet.net (C.-H.L.); lint6494@gmail.com (T.L.); pschou@ym.edu.tw (P.C.); 2 Institute of Public Health, School of Medicine, National Yang-Ming University, Taipei 11221, Taiwan; 3 Department of Medical Research, Taichung Veterans General Hospital, Taichung 40705, Taiwan; 4 Department of Healthcare Management, National Taipei University of Nursing and Health Sciences, Taipei 11219, Taiwan; 5 Department of Emergency Medicine & Orthopedic Surgery, Keelung Hospital, Ministry of Health & Welfare, Keelung 20147, Taiwan

**Keywords:** health, insurance, utilization, emergency department, HIV

## Abstract

It is important that the utilization of emergency departments (EDs) among people living with the human immunodeficiency virus (HIV) be epidemiologically evaluated in order to assess and improve the HIV care continuum. All participants newly-diagnosed with HIV in Taiwan registered in the National Health Insurance Database from 2000 to 2005 were enrolled in this study and followed-up from 2006 to 2011. In total, 3500 participants newly-diagnosed with HIV in 2000–2005 were selected as a fixed-cohort population and followed-up from 2006 to 2011. Overall, 704, 645, 591, 573, 578, and 568 cases made 1322, 1275, 1050, 1061, 1136, and 992 ED visits in 2006, 2007, 2008, 2009, 2010 and 2011, respectively, with an average number of ED visits ranging from 1.75 to 1.98 per person, accounting for 20.1–22.6% of the whole HIV-positive population. Fewer ED visits were due to traumatic reasons, accounting for 19.6–24.4% of all cases. The incidence of traumatic and non-traumatic ED visits among the HIV-positive participants ranged from 7.2–9.3 and 27.0–33.9 per 100 people, respectively. The average direct medical cost of traumatic and non-traumatic ED visits ranged from $89.3–112.0 and $96.6–120.0, respectively. In conclusion, a lower incidence of ED visits for all reasons and fewer ED visits owing to traumatic causes were observed in the population living with HIV in comparison with the general population; however, the direct medical cost of each ED visit owing to both traumatic and non-traumatic causes was greater among those living with HIV than in the general population.

## 1. Introduction

The human immunodeficiency virus (HIV) care continuum was established to improve the population’s understanding of the HIV epidemic as a whole. The spectrum of commitment to the care of persons living with HIV focuses on test-and-treat strategies in particular [1]. In the USA, a 25-year HIV epidemic study including 18,144 adults was performed between 1987 and 2013; the HIV-positive sero-prevalence was 5.2% in 1987; this increased to more than 11% from 1992 to 2003, then diminished to 5.6% in 2013. Among the HIV-positive subjects, the proportion of those with an undiagnosed infection was 77% in 1987, 28% in 1992, and 12% in 2013 (*p* < 0.001) [2]. Based on follow-up of the same cohort as enrolled in the aforementioned study, of the subjects who tested positive for HIV in 2007, 43% were enrolled in care, 39% were retained in care, 27% were receiving anti-retroviral treatment, 26% were aware of their receipt of antiretroviral treatment, 22% were virally-suppressed, and 9% were aware of their viral suppression [3]. However, people living with HIV, either disconnected from or regularly engaged in medical care, commonly visit emergency departments (EDs) with non-HIV-related illnesses or owing to the progression of HIV. Thus, ED HIV care symbolizes the field of HIV prevention, diagnosis, and care [4].

It is important to epidemiologically evaluate the utilization of EDs among the HIV-positive population in order to inform and improve the HIV care continuum, especially in developing countries. The present study was designed in Taiwan, an Asian developing country, with the following aims: (1) to study the trend in ED utilization of the population living with HIV, especially newly-diagnosed patients; (2) to compare the differences in the numbers and types of ED visits (i.e., due to traumatic causes, defined by diagnostic codes including one or more of 20 categories of injury [5], or due to non-traumatic causes, defined by diagnostic codes without any injury) made by persons living with HIV with those of the general population; and (3) to make useful recommendations to Taiwan’s policymakers based on the findings of the study.

## 2. Methods

### 2.1. Data Source, Security, and Quality Control

In Taiwan, a single-payer National Health Insurance (NHI) program was launched in 1995, financed jointly by payroll taxes, subsidizations, and individual payments; the total population of Taiwan is 23.43 million, and the NHI coverage rate is 99.9%. All beneficiaries of NHI are enrolled in the NHI Research Database (NHIRD) [6], which consists of nationwide population-based data with good quality control and representation. The NHIRD is provided to researchers in Taiwan for various investigation purposes [5,7,8,9,10,11]. The health data contained in the NHIRD are scrambled using a double-scrambling procedure in order to protect the confidentiality of patients and caregivers, and, theoretically, it is impossible to probe the data to identify individuals. All academics who wish to use the NHIRD are required to sign a written agreement to declare that they have no intention of attempting to obtain information that could hypothetically violate the confidentiality of patients or healthcare providers.

The present study was a retrospective follow-up analytic study of health insurance data. The protocol was assessed by the NHI Administration, who gave their agreement to the planned analysis of the NHIRD. The data security arrangements and the study plan were also ratified by the Institutional Review Board (IRB) of Taichung Veterans General Hospital, which has been certificated by the Ministry of Health and Welfare, Taiwan.

### 2.2. Definition of the Enrolled Study Population

In this retrospective fixed-cohort study, we examined the health insurance records of 23 million beneficiaries registered in the NHIRD from 2000 to 2005, and enrolled all participants newly-diagnosed with HIV based on the International Classification of Diseases, Ninth Revision, Clinical Modification (ICD-9-CM) diagnostic codes 042.X (HIV infection) and V08.X (asymptomatic HIV infection). The enrolled participants were followed-up from 2006 to 2011 in order to perform an epidemiological study of ED utilization due to different causes among the population living with HIV in Taiwan. To calculate the exact utilization of EDs by those living with HIV, the mortality status of all the participants was considered; valid cases were defined as all participants who were alive on 1 January, and expired cases as all participants who had died before 31 December in each calendar year from 2006 to 2011. The causes of ED visits were categorized into two groups: traumatic (ICD-9-CM diagnostic codes including at least one of 20 categories of injury, ranging from 800.X to 959.X, classified into fracture of the skull (intracranial injury), fracture of the spine or trunk, fracture of an upper limb, fracture of a lower limb, dislocation, sprain and strain of joints and muscles, internal injury of the chest, internal injury of the abdomen or pelvis, open wound of the head/neck/trunk, open wound of an upper limb, open wound of a lower limb, injury to blood vessels, late effects of injuries or other external causes, superficial injury, contusion with intact skin surface, crush injury, effects of a foreign body entering through an orifice, burns, injury to nerves or the spinal cord, and certain traumatic complications/unspecified injuries [5]); and non-traumatic (ICD-9-CM diagnostic codes without any injury). Each ED visit made by the study participants was analyzed in order to assess the annual incidence of ED visits and the direct medical cost per ED visit. Owing to the lack of a control group selected from the general population, the present study was designed solely as a descriptive epidemiological study. Although the annual incidence of ED visits and the direct medical cost were calculated in respective years in a similar way to six cross-sectional surveys, the time trends of the two indicators were estimated as in a cohort study.

### 2.3. Statistical Analysis

Descriptive statistics are represented by numbers of cases, percentages, and means with standard deviation (SD). The independent *t*-test was used to analyze significant differences between groups for continuous variables. 95% confidence intervals (CIs) were calculated, and the Cochran-Armitage trend test was used to identify time trends. All analyses of data were conducted using the Statistical Package for Social Sciences for Windows (SPSS Ver. 22.0, Armonk, New York, NY, USA).

### 2.4. Ethical Approval

The data protection and permission protocols were approved (IRB TCVGH No.: CE13151B-3) by the Institutional Review Board (IRB) of Taichung Veterans General Hospital, which has been certificated by the Ministry of Health and Welfare, Taiwan.

## 3. Results

**Figure 1:**
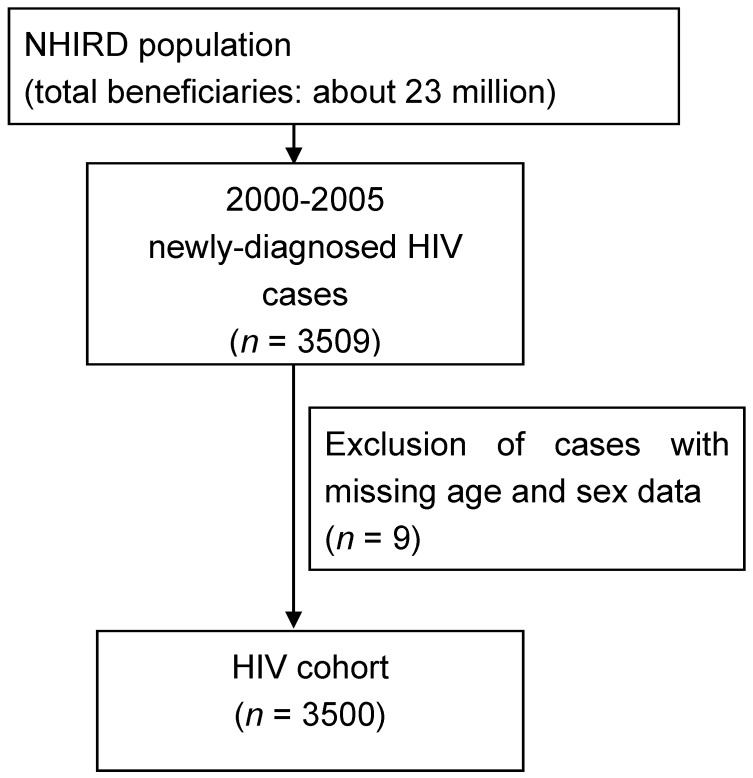
Flow chart of the study population selection.

All 23 million beneficiaries registered in Taiwan’s NHIRD were screened in order to identify participants newly-diagnosed with HIV from 2000 to 2005. The inclusion criterion was an ICD-9-CM diagnostic code of 042.X (HIV infection) or V08.X (asymptomatic HIV infection). In total, 396, 390, 520, 505, 691, and 998 participants were newly-diagnosed with HIV in 2000, 2001, 2002, 2003, 2004, and 2005, respectively; 3500 participants overall were enrolled in the present study as the initial cohort population (as shown in Figure 1). In Table 1, the detailed characteristics of the study population are presented; 90.7% of the HIV-positive population were male, and most were of a young age (46% of the participants were aged 30–44 years; the mean age was 35.1).

The enrolled cases selected as the fixed-cohort population in 2005 were followed-up from 2006–2010. Excluding cases in which data were missing, the number of people living with HIV and the number who had been confirmed as having died were calculated separately (as shown in Table 2). The mortality rate of the population analyzed in the present study ranged from 2.2 to 4.3%. The greatest mortality rate was observed in 2008, which was significantly higher than the rates in the other years studied. Most of the expired persons living with HIV were male, and their mean age ranged from 34.8 to 37.1 years.

To evaluate the utilization of EDs due to traumatic and non-traumatic causes among the population living with HIV in Taiwan, all recorded ED visits of the valid study cases were investigated. As shown in Table 3, 704, 645, 591, 573, 578, and 568 cases made 1322, 1275, 1050, 1061, 1136, and 992 ED visits in 2006, 2007, 2008, 2009, 2010, and 2011, respectively, with an average number of ED visits ranging from 1.75 to 1.98 per person, accounting for 20.1–22.6% of the whole valid HIV-positive study population. Focusing on a comparison of ED visits for traumatic and non-traumatic reasons, fewer ED cases arose for traumatic reasons, accounting for 19.6–24.4% of all cases, and the estimated annual incidence and average direct medical cost per case were calculated. The incidence of traumatic and non-traumatic ED visits among the valid HIV-positive participants ranged from 7.2–9.3 and 27.0–33.9 per 100 people, respectively. The average direct medical cost of traumatic and non-traumatic ED visits ranged from $89.3–112.0 and $96.6–120.0, respectively. A significant increasing trend in the direct medical cost of non-traumatic ED visits was observed.

## 4. Discussion

With an increasing prevalence of HIV, the number of ED visits made by HIV-positive people is increasing globally. A study of 951 HIV-positive cases who participated in face-to-face interviews at 14 HIV clinics in the HIV Research Network, USA aimed to investigate their ED visits, and revealed that 32% of the respondents had made at least one ED visit in the last six months, and more ED visits were made for the management of injuries or other illnesses not related to HIV [12]. Of 107 HIV-positive cases enrolled in a prospective study in the USA, 36% were noted to have received inadequate HIV care (IHC); however, IHC did not predict a greater frequency of ED visits [13]. In the USA, the estimated annual ED visit rate was 633 per 1000 persons known to be living with HIV in 2009 and 438 per 1000 persons living without HIV in 2010 [14]. The present study based in Taiwan showed that between 2006 and 2011, the incidence of traumatic and non-traumatic ED visits among the HIV-positive participants ranged from 7.2–9.3 and 27.0–33.9 per 100 people, respectively, and the incidence of ED visits for any reason in the population living with HIV ranged from 35.7 to 42.7 per 100 people, which was lower than the abovementioned numbers of visits made in the USA between 2009 and 2010. However, there are many differences between the USA and Taiwan, including geographic variation, differences in population sizes, differing race distributions, different healthcare environments and medical payment systems, differing socio-welfare systems, etc. For example, in the USA, the Ryan White Program (RWP) (formerly the Ryan White Comprehensive AIDS Resources Emergency (CARE) Act) was first passed by the US Congress in 1990; Congress has subsequently reauthorized the legislation four times since its initial passage, in 1996, 2000, 2006, and 2009, providing funding for the provision of HIV-related services to the economically-disadvantaged [15]. A study using data from population-based samples of persons receiving care for HIV in three US states revealed that the quality of HIV care provided to patients in RWP-supported facilities was of equivalent or better quality than care provided in non-RWP-supported facilities [16]. Furthermore, in the USA, at most EDs, various health professionals, such as social workers, are allied and can interact with people living with HIV who are seeking treatment primarily for social reasons, such as mental health disorders, addictions, intimate partner violence, child abuse, homelessness, etc., or who may be particularly susceptible to these social issues because of HIV, or owing to the poverty that is associated with HIV. In comparison, medical treatment only is provided at EDs in Taiwan, and it is therefore reasonable that the incidence of ED visits is higher in the USA than in Taiwan.

Some studies have been performed to investigate the use of ED resources among persons living with HIV and the associations with other conditions of social inequity. For example, 428 community-based HIV-positive cases who injected drugs were prospectively followed-up, among whom the cumulative incidence of ED utilization was 63.7% (95% CI 59.1–68.3%) at 12 months after enrollment. The independent factors identified as being associated with the length of time to the first ED visit were unstable housing and an inability to obtain the required healthcare services [17]. In another study that controlled for individual- and community-level factors, ex-prisoner ED visits were found to be significantly more likely to be made for mental health disorders, substance use disorders, and ambulatory care sensitive conditions [18]. A retrospective HIV cohort study of data linked to a hospital discharge dataset was performed during 2007–2010, and revealed that more ED visits were associated with a younger age, the female gender, AIDS-defining illness, AIDS > 1 year, and indigent/charity recipients [19]. Another recent systemic review of studies showed that among people living with HIV/AIDS, a poorer housing status was independently associated with poorer ambulatory medical care outcomes, after controlling for a range of individual case- and care-system-related characteristics [20]. These issues still require further investigation in Taiwan.

Another study that enrolled 28,043 eligible ED patients and another 29,925 eligible patients concluded that non-targeted opt-out rapid HIV screening in EDs was associated with the identification of a modestly-increased number of cases with HIV, the prevalence of new HIV diagnoses in the opt-out phase and the diagnostic phase being 0.05% and 0.01%, respectively [21]. Trauma events are also an important issue in HIV care. A review article revealed higher, but variable, rates of trauma in persons living with HIV, as has been demonstrated in multiple studies, with rates ranging from 10 to 90% [22]. In the present study, all of the enrolled cases were identified as being HIV-positive; further studies are needed in Taiwan to perform comparisons of HIV-positive cohorts with the general population and to identify issues related to injury prevention and social protection programs.

Various studies have focused on the utilization of EDs in Taiwan, one of which revealed that of 1 million beneficiaries, 170,475 subjects had used an ED service in 2010, and 60.5%, 22.3%, 8.7%, and 8.5% of the enrolled patients made 1, 2, 3, 4, and 12 or more ED visits, respectively. People who are admitted to hospital and who make frequent outpatient visits are more likely to have an increased frequency of ED visits, and people with a history of various comorbidities are also more likely to become frequent ED users [23]. In another study based on the same NHIRD from 2000–2004, injury/poisoning was found to be the most frequent diagnostic category recorded in Taiwan’s EDs, accounting for approximately 26.4% of all diagnostic codes. In Taiwan, on average, the treatment-associated cost and drug-associated cost in EDs were reported to be US$35.0 (NT$1155) and US$5.8 (NT$190), respectively, which constitute 64.5% and 10.6% of the total ED-associated expenditure. This means that the ED-related direct medical cost is approximately $55 per person in the general population in Taiwan [24]. 

In the present study, ED visits for traumatic reasons accounted for 19.6–24.4% of all cases, and the average direct medical cost of traumatic and non-traumatic ED visits among the population living with HIV ranged from $89.3–112.0 and $96.6–120.0, respectively; fewer ED visits were made due to traumatic reasons, and the costs of both traumatic and non-traumatic visits made by the HIV-positive cases enrolled in this study were identified as being higher than those in the general population in Taiwan. Furthermore, a significant annual increasing trend in the cost of treatment in EDs for non-traumatic cases was observed in the present study. In Taiwan, ED overcrowding is an important healthcare issue; a total of 12,472,601 ED visits [25] were made by 23,198,664 beneficiaries [26] in 2011, and the estimated incidence of ED visits for all reasons was 53.8 per 100 people in the general population. In the present study, the incidence of ED visits for both traumatic and non-traumatic reasons in the population living with HIV was 35.7–42.7 per 100 people between 2006 and 2011, which was lower than in the general population. However, in Taiwan, nearly 15% of all ED visits were found to be non-emergency visits, and an additional 20% were identified as being emergency-preventable through primary care in the general population [27]. Furthermore, a more recent study concluded that in Taiwan, non-emergency ED visits may be reduced by increasing the availability of ambulatory care facilities in areas deficit in such facilities, but are not reduced in areas with medium to high availability of ambulatory care facilities [9]. 

From the viewpoint of national HIV screening policy, EDs are important places in which ED physicians, nurses, and social workers can promote HIV screening programs, and obtain informed consent for all eligible patients [28]. In the present study, it was found that people living with HIV in Taiwan, who make fewer ED visits and have a higher ED cost per visit than the general population, may present with complex problems secondary to discrimination (e.g., stigma), social inequity (e.g., poverty, marginalization), insufficient local medical resources (e.g., social worker support, community nursing care), and more complicated medical illnesses or injuries that require more comprehensive assessment, treatment, and even referral. Furthermore, some HIV prevention strategies have also been considered by the authorities in Taiwan. For example, there has been a positive impact of news trends on online search behavior in terms of HIV-related issues [29]; in addition, pre-exposure prophylaxis (PrEP) has become an important part of more recent HIV prevention programs, especially those that focus increasingly on biomedical prevention technologies [30]. Policymakers in Taiwan should be aware that strengthening the healthcare system for the HIV-positive population is necessary, and the findings of the present study may also apply to other nearby Asian nations.

## 5. Conclusions

Overall, 20–23% of the persons living with HIV enrolled in the present study used ED resources, with an average number of ED visits ranging from 1.75 to 1.98 per person annually, and the incidence of ED visits owing to traumatic and non-traumatic causes among the HIV-positive population ranged from 7.2–9.3 and 27.0–33.9 per 100 people, respectively. A lower incidence of ED visits for all reasons and fewer ED visits due to traumatic causes were noted in the population living with HIV in comparison with the general population; however, the direct medical cost of each ED visit among the HIV-positive participants, for both traumatic and non-traumatic reasons, was higher than that in the general population.

## Figures and Tables

**Table 1:** Dynamic inclusive cohort of people newly-diagnosed with HIV * enrolled in the present study in Taiwan during 2000–2005.

Variables	Identified in 2000 (*n* = 396)		Identified in 2001 (*n* = 390)		Identified in 2002 (*n* = 520)		Identified in 2003 (*n* = 505)		Identified in 2004 (*n* = 691)		Identified in 2005 (*n* = 998)		Cumulative Enrolled Cases (*n* = 3500)	
	No.	%	No.	%	No.	%	No.	%	No.	%	No.	%	No.	%
**Age, mean (SD)**	35.7 (11.9)		36.1 (11.6)		36.6 (11.9)		35.1 (12.2)		34.9 (12.1)		34.0 (10.6)		35.1 (11.6)	
0–29 years	146	36.9	123	31.5	166	31.9	205	31.9	260	37.6	412	41.3	1312	37.5
30–44 years	169	42.7	197	50.5	254	48.9	217	43.0	315	45.6	456	45.7	1608	45.9
45–59 years	64	16.2	54	13.9	66	12.7	59	11.7	80	11.6	100	10.0	423	12.1
60+ years	17	4.3	16	4.1	34	6.5	24	7.6	36	5.2	30	3.0	157	4.5
**Gender**														
Female	41	10.4	33	8.5	36	6.9	37	7.3	63	9.1	115	11.5	325	9.3
Male	355	89.6	357	91.5	484	93.1	468	92.7	628	90.9	883	88.5	3175	**90.7**

* HIV-positive cases: ICD-9-CM diagnostic codes 042.X (HIV infection) and V08.X (asymptomatic HIV infection).

**Table 2:** Valid cases enrolled in 2005 and followed-up from 2006–2013.

	2006	2007	2008	2009	2010	2011
	No. (%)	No. (%)	No. (%)	No. (%)	No. (%)	No. (%)
Valid cases *	3113	3021	2944	2817	2754	2676
Expired cases **	92	77	127	63	78	72
Male (%)	81 (88.0)	65 (84.4)	113 (89.0)	59 (93.7)	70 (89.7)	67 (93.1)
Age, mean (SD) (years)	36.2 (14.0)	36.7 (11.5)	36.5 (11.4)	34.9 (10.4)	34.8 (11.0)	37.1 (13.8)
Mortality rate (95% CI) ***	3.0% (2.4–3.6)	2.5% (2.0–3.2)	4.3% (3.6–5.1)	2.2% (1.8–2.9)	2.8% (2.3–3.5)	2.7% (2.1–3.4)

* Valid cases: all cases who were alive on 1 January in the respective year. ** Expired cases: all cases who had died before 31 December in the respective year. *** 95% CI: 95% confidence interval.

**Table 3:** Trends in and comparisons of emergency department (ED) utilization in the studied population.

	2006	2007	2008	2009	2010	2011	*p* Value for Trend *
Total ED visitors	704	645	591	573	578	568	
ED utilization rate (visitors/valid cases)	22.6%	21.4%	20.1%	20.3%	21.0%	21.2%	
Frequency of ED visits with traumatic-related * causes (% of all visits)	291 (21.8)	252 (19.8)	256 (24.4)	242 (22.8)	237 (20.9)	194 (19.6)	
Incidence of traumatic ED visits among the valid cases, per 100 persons (95% CI)	9.3 (8.4–10.4)	8.3 (7.4–9.4)	8.7 (7.7–9.8)	8.6 (7.6–9.7)	8.6 (7.6–9.7)	7.2 (6.3–8.3)	0.17
Frequency of ED visits with non-traumatic-related causes (% of all visits)	1041 (78.2)	1023 (80.2)	794 (75.6)	819 (77.2)	899 (79.1)	798 (80.4)	
Incidence of non-traumatic ED visits among the valid cases, per 100 persons (95% CI)	33.4 (31.8–35.1)	33.9 (32.2–35.6)	27.0 (25.4–28.6)	29.1 (27.4–30.8)	32.6 (30.9–34.4)	29.8 (28.1–31.6)	0.39
Medical Cost (US$) **, mean (SD)							
Traumatic cases	92.1 (93.5)	96.4 (106.0)	104.0 (142.0)	112.0 (214.0)	93.9 (107.0)	89.3 (102.0)	0.90
Non-traumatic cases	96.6 (143.0)	102.0 (121.0)	104.0 (153.0)	103.0 (146.0)	105.0 (176.0)	120.0 (179.0)	0.005
*p * value for *t* test	0.52	0.26	0.99	0.55	0.21	0.002	

* Cochran-Armitage trend test. ** USD: NTD = 1:32.
